# Huygens' Principle geometric derivation and elimination of the wake and backward wave

**DOI:** 10.1038/s41598-021-99049-7

**Published:** 2021-10-12

**Authors:** Forrest L. Anderson

**Affiliations:** Unaffiliated, Tucson, USA

**Keywords:** Physics, Optical physics

## Abstract

Huygens' Principle (1678) implies that every point on a wave front serves as a source of secondary wavelets, and the new wave front is the tangential surface to all the secondary wavelets. But two problems arise: portions of wavelets that exist outside of the new wave front combine to form a wake. Also there are two tangential surfaces so wave fronts are propagated in both the forward and backward directions. These problems have not previously been resolved by using a geometrical theory with impulsive wavelets that are in harmony with Huygens' geometrical description. Doing so would provide deeper understanding of and greater intuition into wave propagation, in addition to providing a new model for wave propagation analysis. The interpretation, developed here, of Huygens' geometrical construction shows Huygens' Principle to be correct: as for the wake, the Huygens' wavelets disappear when combined except where they contact their common tangent surfaces, the new propagating wave fronts. As for the backward wave, a source propagates both a forward wave and a backward wave when it is stationary, but it propagates only the forward wave front when it is advancing with a speed equal to the propagation speed of the wave fronts.

## Introduction

**Figure 1:**
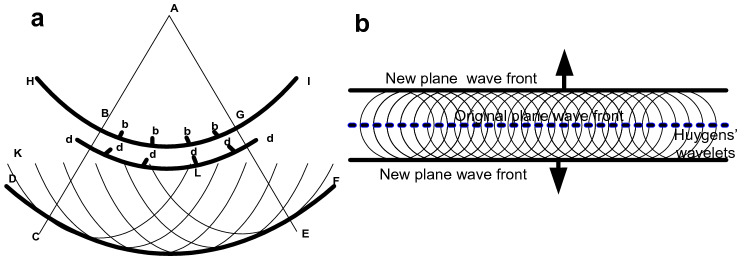
Huygens' Principle as a geometrical construction. (**a**) A replica of Huygens' original Figure1 (a spherical wave). (**b**) Huygens' geometrical construction presented as a plane wave.

Huygens' Principle implies that every point on a wave front serves as a source of secondary wavelets. The new wave front is the tangential surface to all the secondary wavelets in the direction of propagation (Fig. 1 presents this as a geometric construction).

An abundance of literature refers to Huygens' Principle^1–3^. References^4–32^ are a very small sample which show the tremendous variation in the application of Huygens' Principle and some continuing work on it. But there has been insufficient theory developed which addresses the underlying Huygens' basic geometrical construction of how a wave propagates via Huygens' wavelets. “At present mathematical physicists are busy with the rapid developments of modern physics, and older problems are likely to be neglected” (referring to Huygens Principle in 1940)^33^.

This is despite the common use of Huygens' construction to introduce wave theory, reflection, refraction, and diffraction. In his construction two problems arise: the portions of the wavelets not contacting the position of the new propagating wave must disappear (‘no wake’)^2, 3^. Also, the portions of the wavelets propagating in the direction opposite to the new propagating wave front must vanish (‘no backward wave’). There has been no clear direct geometrical theory with impulsive wavelets that are in harmony with Huygens' geometrical description which shows how this can occur.

Huygens' Principle's problems of the backward wave and the wake have been approached before by relying on physics (for example, suitable initial values of the velocity potential and condensation^3^), or by involving doublets, dipoles, obliquity factors, sinusoidal waves, and also by using complex mathematics^2, 3^, “..but the intuitive appeal of Huygens' simple principle is lost...”^4^ along with a clear direct connection to his geometrical construction and the physical insight that derives from it.

Wave propagation is linear so superposition holds: it should be possible to decompose an impulsive propagating wave front into its constituent points, then consider the impulsive wavelets radiating from each of those points at a future time, and combine those wavelets in a simple direct geometric manner to obtain the progressing wave front at that future time.

The following shows that Huygens' geometrical construction is correct as depicted in his figures when the wavelets are interpreted as Dirac Delta distributions (Delta functions)^34–38^, and when the propagating wave field resulting from the summation of the wavelets is differentiated to yield the wave front, and also when the speed of the source is taken into account. Given this, the backward wave front and wake both vanish.

The following development is geometric and does not involve the wave equation or any particular type of field (except in the Supplementary Information Notes). The symbol *φ* is used for the propagating wave fields and *f* is used for wave fronts derived from *φ*. The symbol *δ* is used for the Dirac delta distribution and *δ*_*f*_ is used for finite approximations to it. The symbol *θ* is used for the Heaviside step function^36, 37^. All results are in the sense of distributions.

## Results

The ensuing analyzes the wave fields and wave fronts from stationary (section “Stationary planar source and elimination of the wake”) and moving (section “Moving planar source and elimination of the backward wave”) impulsive infinite planar sources and also nonexpanding (section “Non expanding spherical source and elimination of the wake”) and expanding (section “Expanding spherical source and elimination of the backward wave”) impulsive spherical sources. This is done because planar and spherical sources are the most fundamental extended wave sources, and the analysis here must work (replicate the original source wave with no wake) for each of them. Also it must produce the correct results (no backward wave) when the sources are moving or expanding at the wave propagation speed.

In addition the planar source analysis supplies necessary results that are required for the spherical source analysis. Also, the planar source results are used to check the spherical source results in the limit as the radius goes to infinity.

Each of the sections “Moving planar source and elimination of the backward wave”, “Non expanding spherical source and elimination of the wake”, “Expanding spherical source and elimination of the backward wave”) depends on, and/or corroborates, results from the preceding sections. In addition the proceeding sections are used to corroborate the preceding sections.

The results from section “Stationary planar source and elimination of the wake” are needed to produce the results in section “Moving planar source and elimination of the backward wave”. (This is because in the limit as the pulse width goes to zero the area of the frustum is the same as the area of the annulus) Also in the limit of no source motion section “Moving planar source and elimination of the backward wave” results corroborate section “Stationary planar source and elimination of the wake” results.

The results from section “Non expanding spherical source and elimination of the wake” are needed to produce the results in section “Expanding spherical source and elimination of the backward wave”. (This is because, again, in the limit as the pulse width goes to zero the area of the frustum is the same as the area of the annulus) Also in the limit of no source expansion section “Expanding spherical source and elimination of the backward wave” results corroborate section “Non expanding spherical source and elimination of the wake” results.

The method used for the moving planar source, section “Moving planar source and elimination of the backward wave”, is used in the expanding spherical source, section “Expanding spherical source and elimination of the backward wave”, to implement the effects of motion on the wavelet point sources initial impulses due to the sphere's radius expanding with a speed *v*: this is possible because, again, in the limit as the pulse width goes to zero the area of the frustum is the same as the area of the annulus.

The notes in the SI are there to corroborate the results in the main text. There are references to each note at the appropriate location in the main text.

An exception, note E geometrically derives the integral term in D'Alemberts formula which is used in the SI This derivation is originated here.

With this I have shown the linkage between the analysis sections “Stationary planar source and elimination of the wake”, “Moving planar source and elimination of the backward wave”, “Non expanding spherical source and elimination of the wake”, “Expanding spherical source and elimination of the backward wave”, and also the linkage with the notes in the SI.

Each major analysis section derives a major conclusion:

Section I: a stationary planar source has no wake.

Section II: a planar source moving at the speed of propagation also has no backward wave.

Section III: a non expanding spherical source has no wake.

Section IV: an spherical source expanding at the speed of propagation also has no backward wave.

Note that the moving sources (planar or spherical) moving at the speed of propagation, c. are in fact the propagating wave fronts populated by secondary sources and mentioned in Huygens’ principle; and are the reason for the backward wave elimination, as will be shown..

### Stationary planar source and elimination of the wake

(The infinite planar source may sometimes be referred to as ‘planar source’ or ‘plane’).

This section analyzes the waves propagating away from a motionless infinite planar source. Each point on the plane is a source of a Huygens' wavelet consisting of a spherical Dirac delta function (distribution) expanding as a function of time at a rate equal to c, the propagation speed. The strength (or amplitude) of the wavelet is α which is attenuated as the wavelet expands by spherical spreading.

**Figure 2:**
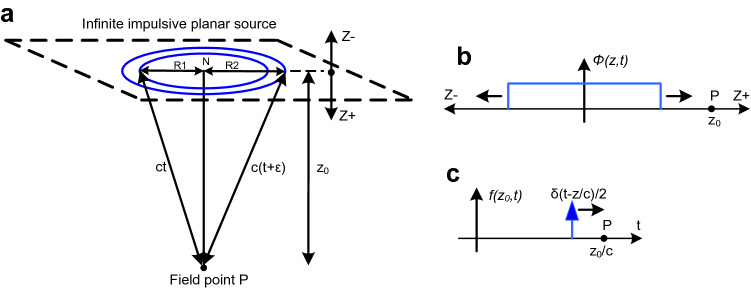
Planar source. (**a**) An infinite plane with impulsive excitation, *δ*_*f*_(*t*). The excitation duration or temporal pulse width is *ε*. The parameter *z*_0_ is the distance from the plane to a point in space, *P*, at which the wave field will be observed as a function of time. The propagation speed is *c*, where *c* > 0. *R*1 and *R*2 are the radii of an annulus defined by the intersection of the plane with two spheres of radii *ct* and *c*[*t* + *ε*]. The point *N* is the nearest point on the plane to the field point *P*. Each point on the plane is a point source which radiates an impulsive spherical wavelet. (**b**) The wave field *ϕ* that is propagated from the plane. (**c**) The wave front *f* in the *Z* + direction that is derived from the wave field which will arrive at *P* at *t* = *z*_0_*/c.*

It is shown that the wave field (summation of the Huygens' wavelets) observed as a function of time at some fixed point is a constant value after the wavelets first reach that point. The partial derivative with respect to time of that wave field gives the wave front which replicates the original wave (the Huygens' wavelet) at 1/2 amplitude in both the advancing and retreating directions. The derivative also shows there is no wake. See Fig. 2.

It is only necessary to derive the wave field for each point on any one line perpendicular to the source since the planar source is uniform with respect to the *x* and *y* coordinates. Only the points in the planar source intersected by the sphere with radius *ct* centered at *P* can contribute to the wave field *φ*(*z*_0_, *t*) when deriving the wave field at *P* at time *t* (Fig. 2a).

Initially the duration *ε* of the excitation pulse *δ*_*f*_ will be finite and the pulse will have an height equal to *α*. Then the area (or strength) under this finite version of *δ*(*t*) is *εcα* which is required to be 1. Later, where needed, the limit as *ε* tends toward zero will be taken while still requiring *α* to take on a value such that *εcα* remains equal to 1 (i.e. *α* = 1*/*[*εc*]) so that *δ*_*f*_ → *δ* as *ε → *0.

The locus of all radiating points on the planar source which can contribute to the wave field *φ*(*z*_0_, *t*) during the time interval [*t*, *t* + *ε*] and at distance *z*_0_ from the plane is an annular area. This annular area is bounded by two concentric spherical shells of radius *ct* and *c*[*t* + *ε*].

The area *Ap* of the annulus is:

1 $$Ap = \pi R2_{{}}^{2} - \pi R1_{{}}^{2} \,$$

where

2 $$R1_{{}}^{2} = c^{2} t^{2} - z_{0}^{2}$$

and

3 $$R2_{{}}^{2} = c^{2} \left[ {t + \varepsilon } \right]^{2} - z_{0}^{2} = c^{2} \left[ {t^{2} + 2t\varepsilon + \varepsilon^{2} } \right] - z_{0}^{2} = c^{2} t^{2} + 2c^{2} t\varepsilon + c^{2} \varepsilon^{2} - z_{0}^{2}$$

So

4 $$Ap = \pi c^{2} \left[ {2t\varepsilon + \varepsilon^{2} } \right]$$

which has the limit

5 $$Ap = 2\pi c^{2} t\varepsilon {\text{ as }}\varepsilon \to {0}$$

(and so *δ*_*f*_ → *δ*).

Spherical spreading attenuation specified to be of the form 1*/*[*4πct*] attenuates the wavelets and therefore the radiation from the annular area. As a result the attenuated radiation from *Ap* is not a function of the field point's distance, *z*_0_, from the planar source (except for a step at *ct* = *z*_0_ as the wave front passes). This will be shown in the following:

Combining the 1*/*[*4πct*] spherical spreading attenuation and the *α* height factor with the area *Ap* gives the (dimensionless) amplitude of the wave field, *φ*, after the impulsive spherical wavelets have all passed (i.e. for *|z|*≤ *ct*), yielding:

6 $$\varphi = Ap\frac{\alpha }{4\pi ct} = \frac{{2\pi c^{2} t\varepsilon }}{4\pi ct}\alpha = \frac{2\pi ct}{{4\pi ct}}c\varepsilon \alpha = \frac{c\varepsilon \alpha }{2}$$

Because *cεα* = 1, the result simplifies to

7 $$\varphi = \frac{1}{2}{\text{ for }}\left| z \right| \le ct$$

or using a Heaviside step function, *θ*,

8 $$\varphi \left( {z,t} \right) = \frac{1}{2}\theta \left( {ct - \left| z \right|} \right)$$

which as a spatial function of *z* is a rectangular pulse expanding in time in both advancing (toward *P*) and retreating (away from *P*) directions (Fig. 2b).

In the advancing direction the wave field observed at *P* as a function of time is

9 $$\varphi \left( {z_{0} ,t} \right) = \frac{1}{2}\theta \left( {ct - z_{0} } \right)$$

which has a transition at *t* = *z*_0_*/c* when the wave field has advanced to *P*.

The derivative of *φ* yields an advancing planar wave front, *f*, which observed at *P* as a function of time is (Fig. 2c):

10 $$f^{ + } \left( {z_{0} ,t} \right) = \frac{\partial }{\partial t}\frac{1}{2}\theta \left( {ct - z_{0} } \right) = \frac{c}{2}\delta \left( {ct - z_{0} } \right) = \frac{c}{2}\delta \left( {c[t - \frac{{z_{0} }}{c}]} \right) = \frac{c}{2c}\delta \left( {t - \frac{{z_{0} }}{c}} \right) = \frac{1}{2}\delta \left( {t - \frac{{z_{0} }}{c}} \right)$$

Or for *z* in general

11 $$f^{ + } \left( {z,t} \right) = \frac{\partial }{\partial t}\frac{1}{2}\theta \left( {t - \frac{z}{c}} \right) = \frac{1}{2}\delta \left( {t - \frac{z}{c}} \right)$$

This holds for every line perpendicular to the source. Note that only the single point source at the nearest point N contributes to the wave front at *P*: the wave field *φ* is a step function and its derivative is zero except at the step's location, and the step was caused by the wave field originating at N (Fig. 2a). (So there is a one to one correspondence between the points in the wave front and the points in the planar source.) Because the derivative is zero except at the step, there is no wake.

There will also be a retreating plane wave front *f* ^*-*^(*z*, *t*) = *½* *δ*(*t* + *z/c*) propagating in the opposite direction, away from *P*, which can be derived similarly. This also propagates without a wake. (Here *z* is negative, and *f* will not be seen at *z*_0_, the location of *P*).

Therefore an infinite planar distribution of point sources radiating impulsive spherical wavelets creates advancing and retreating impulsive plane wave fronts that propagate cleanly without a wake. This shows that the Huygens' wavelets cancel each other when summed together and differentiated except on their common tangential surfaces (Fig. 1a,b).

It might be expected that the previous process can be repeated and the impulsive planar wave fronts made to progress to the next future time *t* + *Δt*, and so on. But because of the ½ factor the waves would be attenuated upon each iteration. When the source is moving at the same speed as the advancing wave front the ½ factor disappears along with the retreating or backward wave front, as will be shown:

### Moving planar source and elimination of the backward wave

**Figure 3:**
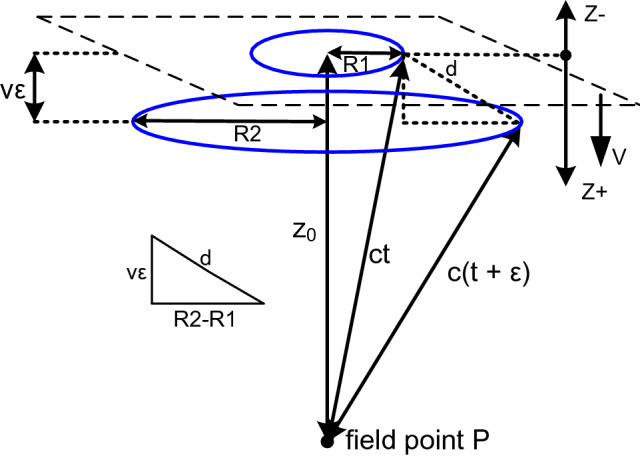
The geometry of an infinite planar impulsive source moving toward *P* with speed *v*. (a speed away from *P* would be given by *−* *v*.) *R*1 is the radius of the circle created by the intersection at time *t* of a sphere of radius *ct* with the plane. *R*2 is the radius of the circle created by the intersection at time *t* + *ε* of a sphere of radius *c*[*t* + *ε*] with the plane.

This section analyzes the changes that occur in the results of section “Stationary planar source and elimination of the wake” when the planar source is moving with speed v toward the observation point. It is shown that the results from section “Stationary planar source and elimination of the wake” can be used if the effects of motion on the Huygens' wavelets are included. When this is done it is seen that a new additional wave is created as a function of the planar source motion, v. This additional wave reduces the amplitude of the backward wave and increases the amplitude of the forward wave. When v = c the backward wave is eliminated and the forward wave amplitude is doubled eliminating the 1/2 factor. In any case the original wave is replicated. See Fig. 3.

During the time interval *ε* the planar source moves a distance *vε* in the direction toward *P*. Then *R*2 is the radius of the base of a right circular cone of slant height *c*[*t* + *ε*] and *R*1 is the radius of the base of a right circular cone of slant height *ct*. These two bases define a conical frustum of height *vε* and slant height *d*. The lateral area of this frustum, *π*[*R*2 + *R*1]*d*, is the locus of all radiating points which can contribute to the field at the field point *P* during the time interval [*t*, *t* + *ε*]. This is analogous to the annular area on the infinite stationary plane previously considered.

However, in the limit of *ε → *0 the area of the frustum reduces to the area of that annulus as follows:

The annulus area is *π*[*R*2^2^ *−* *R*1^2^] and the frustum area is *π*[*R*2 + *R*1]*d*. Then referring to Fig. 3, *d*^2^ = *v*^2^*ε*^2^ + [*R*2 *−* *R*1]^2^, and as *ε → *0, *d*^2^ = [*R*2 *−* *R*1]^2^. So *d* = *R*2 *−* *R*1.

Therefore as *ε → *0 the frustum area becomes *π*[*R*2 + *R*1]*d* = *π*[*R*2 + *R*1][*R*2 *−* *R*1] = *π*[*R*2^2^ *−* *R*1^2^], which is also the area of the annulus.

**Figure 4:**
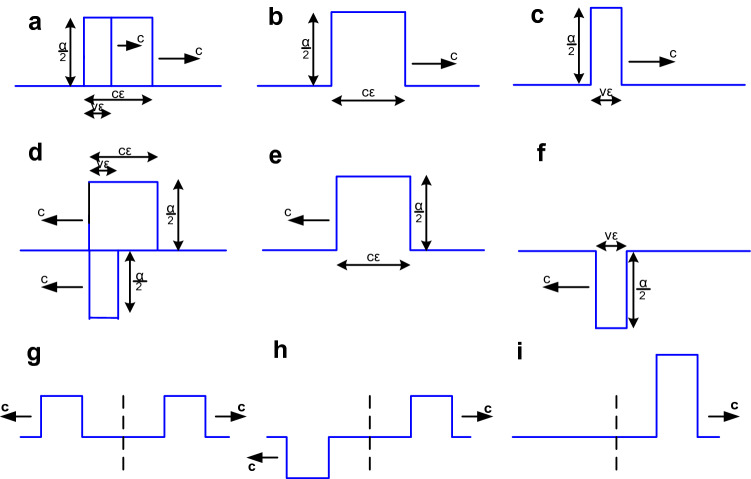
(**a–c**) Source motion displacement for the forward wave: (**a**) the original forward wave front pulse compared with the additional forward wave front pulse due to the source motion. (**b**) The original forward wave front pulse which is the same as the wave front pulse from the stationary source. (**c**) The additional forward wave front pulse due to the source motion. (**d**–**f**) Source motion displacement for the backward wave: (**d**) the original backward wave front pulse compared with the additional backward wave front pulse due to the source motion. (**e**) The original backward wave front pulse which is the same as the wave front pulse from the stationary source. (**f**) The additional backward wave front pulse due to the source motion. (**g**–**i**) The original and additional wave fronts with *v* = *c*. (**g**) Forward and backward wave front pulses that would propagate from a stationary source. (**h**) Additional wave front pulses originating from source motion. (**i**) Forward wave front pulse created by the sum of the other two wave front pulses (**g**) and (**h**).

Consequently the previously developed results and formulas for the infinite stationary plane can be used. However, the effects of the motion of the moving source on its point sources' initial radiated impulses must still be considered:

See Fig. 4a–c. If the planar impulsive source is moving with positive source speed, *v*, toward *P*, the motion results in a displacement during the pulse time interval ε (an additional initial condition) which causes additional wave front pulses to propagate in both the forward and backward directions, as with the stationary planar source. (Displacement at *x* is approximately the product of the temporal integration interval and the displacement speed evaluated at *x.* See Supplementary Notes B and E) This is in addition to the previously derived wave front pulses from the stationary planar impulsive source. The displacement height is the same as the original forward wave front pulse, *α/*2, whether longitudinal or transverse. The displacement spatial extent is *vε.*

The source motion in the forward direction displaces a positive *vεα/*2 during the time interval *ε* which propagates forward as an additional wave front pulse. Since *α* = 1*/*[*cε*] the displacement or strength equals [1*/*2][*vε*]*/*[*cε*] = *v/*2*c*. Do to linearity this additional wave front pulse is added to the original stationary planar source wave front pulse. Note that as *ε → *0 a pulse of width *ε* and height 1*/ε* moving at speed *c* tends to *δ*(*x* *−* *ct*). Note also that *v* and *c* are scalars and do not have to be measured along the same direction–they effect pulse strength by different means.

Then the total resulting wave front in the forward direction is the sum of two wave front pulses which, as *ε → *0, can be combined to yield:

12 $$f^{ + } \left( {z,t} \right) = \frac{1}{2}\delta \left( {t - \frac{z}{c}} \right) + \frac{v}{c}\frac{1}{2}\delta \left( {t - \frac{z}{c}} \right) = \left[ {1 + \frac{v}{c}} \right]\frac{1}{2}\delta \left( {t - \frac{z}{c}} \right) = \frac{v + c}{{2c}}\delta \left( {t - \frac{z}{c}} \right)$$

See Fig. 4d–f. Proceeding similarly with the equalizing additional backward wave where the displacement is − *vεα* since the propagation direction is opposite to the source motion direction. The source motion displaces an equalizing negative − vεα/2 during the pulse time interval *ε* which then propagates backward as an additional wave front pulse. Since *α* = 1*/*[*cε*] the displacement or strength equals [1*/*2][*−* *vε*]*/*[*cε*] = *−* *v/*2*c*. As before this wave front pulse is added to the original stationary planar source wave front pulse. Then the total resulting wave front in the backward direction (where *z* is negative) is the sum of two wave front pulses which, as *ε → *0, can be combined to yield:

13 $$f^{ - } \left( {z,t} \right) = \frac{1}{2}\delta \left( {t + \frac{z}{c}} \right) + \frac{ - v}{c}\frac{1}{2}\delta \left( {t + \frac{z}{c}} \right) = \left[ {1 + \frac{ - v}{c}} \right]\frac{1}{2}\delta \left( {t + \frac{z}{c}} \right) = \frac{ - v + c}{{2c}}\delta \left( {t + \frac{z}{c}} \right)$$

The sign is negative for the additional wave front pulse that is propagating in the direction opposite to the motion of the source (in the backward direction). This relative direction of motion defines the terms ‘forward’ and ‘backward’ in the case of the moving planar impulsive source. (Note that the terms are not defined for a stationary planar impulsive source).

So the impulsive forward and backward planar wave fronts are

14 $$f^{ + } \left( {z,t} \right) = \frac{v + c}{{2c}}\delta \left( {t - {z \mathord{\left/ {\vphantom {z c}} \right. \kern-\nulldelimiterspace} c}} \right){\text{ and }}f^{ - } \left( {z,t} \right) = \frac{ - v + c}{{2c}}\delta \left( {t + {z \mathord{\left/ {\vphantom {z c}} \right. \kern-\nulldelimiterspace} c}} \right)$$

(At *P* at a positive distance along the z axis, only the forward wave front will be observed.) For the stationary planar source (where *v* = 0), we get *f*^+^(*z*,*t*) = *½* *δ*(*t* *−* *z/c*) for the forward wave front and *f*^*−*^(*z*,*t*) = *½* *δ*(*t* + *z/c*) for the backward wave front, as was derived before.

See Fig. 4g–i. For source speed *v* = *c* the forward wave front is *f*^+^(*z*, *t*) = *δ*(*t* *−* *z/c*) or as seen at *P* as a function of time, *f*^+^(*z*_0_, *t*) = *δ*(*t* *−* *z*_0_*/c*). So the forward wave front propagates without changing and becomes the new progressing Huygens' wave front (Note the absence of the ½ factor). For the backward wave front *f* ^*-*^(*z*,*t*) = 0. So there is no backward wave front (See SI Notes C, D).

### Non expanding spherical source and elimination of the wake

This section analyzes the waves propagating away from a non expanding spherical source. Each point on the sphere is a source of a Huygens' wavelet consisting of a spherical Dirac delta function (distribution) expanding as a function of time at a rate equal to c, the propagation speed. The strength (or amplitude) of the wavelet is α which is attenuated as the wavelet expands by spherical spreading.

**Figure 5:**
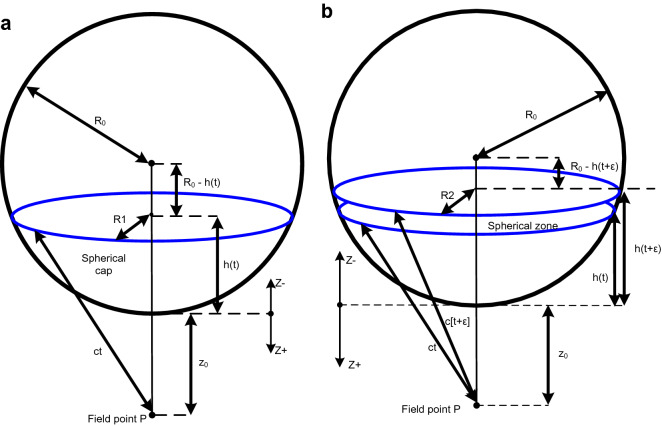
Non expanding spherical shell impulsive source. (**a**) A spherical cap on an impulsive spherical shell source. (The spherical shell may be referred to as a 'sphere' hereafter.) The radius is *R*_0_, and the sphere is centered at the coordinate origin and has impulsive excitation *δ*_*f*_ (*t*). Each point on the sphere’s surface represents a point source which radiates an impulsive spherical wavelet. The parameter *z*_0_ is the radial distance from the sphere's surface to the point in space, *P*, at which the resultant wave field *φ* will be observed as a function of time. The propagation speed is *c*. The distance *ct* from *P* to a point on the sphere defines a spherical cap of height *h*(*t*) with radius *R*1. The sphere is transparent to radiation so that the whole sphere will contribute to the wave field at any one point in space. (**b**) The locus of all contributing radiating points is a spherical zone. These are the only points on the spherical source of radius R_0_ which can contribute to the field at *P* during the time interval [*t*, *t* + *ε*] and at distance *z*_0_ from the sphere. The zone on the spherical source is bounded by its intersection with two concentric spheres of radius *ct* and radius *c*[*t* *−* *ε*] centered at the field point *P*. The intersections defines spherical caps of heights *h*(*t*) and *h*(*t* + *ε*), also radii *R*1 and *R*2.

It is shown that the wave field (summation of the wavelets) observed as a function of time at some fixed point is a constant value after the wavelets first reach that point and remain constant for a period of time that is proportional to the sphere's diameter. The partial derivative with respect to time of that wave field gives the wave front which replicates the original wave (the Huygens' wavelet) at 1/2 amplitude in both the expanding and converging directions. That derivative also shows there is no wake. See Fig. 5.

Since the spherical source is symmetrical, it is only necessary to derive the wave field for each point on any one radial line emanating from its center. The wave field will be the same on any other radial line. As before, initially it is assumed that the temporal duration *ε* of the excitation pulse *δ*_*f*_ is finite and that it has a height equal to *α* and an area equal to *εcα*, where *α* = 1*/*[*εc*] to maintain an area equal to 1 as *ε → *0 (and *δ*_*f*_ → *δ*).The surface area of a spherical cap is 2*πR*_0_*h*(*t*) (Fig. 5a), and the surface area of the spherical zone is *As* = 2*πR*_0_[*h*(*t* + *ε*) *−* *h*(*t*)] (Fig. 5b); Note surprisingly that the surface area of the zone depends only on its height, *h*(*t* + *ε*) *−* *h*(*t*), not on its vertical position within the sphere.

To find *As* the heights *h*(*t*) and *h*(*t* + *ε*) must be found. Two right triangles can be formed (Fig. 5a):

15 $${\text{T1: }}c^{2} t^{2} = R1^{2} + \left[ {h(t) + z_{0} } \right]^{2} {\text{ and }}$$

16 $${\text{T2: }}R_{0}^{2} = R1^{2} + \left[ {R_{0} - h(t)} \right]^{2}$$

Solving for *h*(*t*) gets

17 $$T1 - T2:c^{2} t^{2} - R_{0}^{2} = \left[ {h(t) + z_{0} } \right]^{2} - \left[ {R_{0} - h(t)} \right]^{2}$$

18 $$c^{2} t^{2} - R_{0}^{2} = h^{2} (t) + 2h(t)z_{0} + z_{0}^{2} - R_{0}^{2} + 2R_{0} h(t) - h^{2} (t)$$

19 $$c^{2} t^{2} - R_{0}^{2} = 2h(t)z_{0} + z_{0}^{2} - R_{0}^{2} + 2R_{0} h(t)$$

20 $$c^{2} t^{2} = 2h(t)z_{0} + z_{0}^{2} + 2R_{0} h(t)$$

21 $$2h(t)\left[ {R_{0} + z_{0} } \right] = c^{2} t^{2} - z_{0}^{2}$$

22 $$h(t) = \frac{{c^{2} t^{2} - z_{0}^{2} }}{{2\left[ {R_{0} + z_{0} } \right]}}$$

and also (by substituting *t* + *ε* for *t*)

23 $$h(t + \varepsilon ) = \frac{{c^{2} \left[ {t + \varepsilon } \right]^{2} - z_{0}^{2} }}{{2\left[ {R_{0} + z_{0} } \right]}}$$

where both equations are for time *t* where *z*_0_*/c* ≤ *t* ≤ [*R*_0_ + *z*_0_]*/c*.

Then the zone's surface area is

24 $$\begin{gathered} As = 2\pi R_{0} \left[ {h\left( {t + \varepsilon } \right) - h\left( t \right)} \right] = 2\pi R_{0} \frac{{c^{2} \left[ {t + \varepsilon } \right]^{2} - z_{0}^{2} - c^{2} t^{2} + z_{0}^{2} }}{{2\left[ {R_{0} + z_{0} } \right]}} \\ \, = 2\pi R_{0} \frac{{c^{2} t^{2} + c^{2} 2t\varepsilon + c^{2} \varepsilon^{2} - z_{0}^{2} - c^{2} t^{2} + z_{0}^{2} }}{{2\left[ {R_{0} + z_{0} } \right]}} = \pi R_{0} \frac{{c^{2} 2t\varepsilon + c^{2} \varepsilon^{2} }}{{R_{0} + z_{0} }} \\ \end{gathered}$$

and as *ε → *0 (so *δ*_*f*_ → *δ*)

25 $$As = \frac{{2\pi R_{0} c^{2} t\varepsilon }}{{R_{0} + z_{0} }} = \frac{{R_{0} }}{{R_{0} + z_{0} }}2\pi c^{2} t\varepsilon$$

which is the same as the area of the stationary infinite plane annulus *Ap* but attenuated by the spherical spreading factor *R*_0_*/*[*R*_0_ + *z*_0_] (the radial lines represent infinitesimal solid angles). This area is also the same as the area of the plane's annulus *Ap* when *R*_0_* → ∞* or when *z*_0_* → *0.

As before spherical spreading of the form 1*/*[*4πct*] attenuates the wavelets and therefore the radiation from the spherical zone. The dimensionless amplitude of *φ* observed at *P* for *z*_0_*/c* ≤ *t* ≤ [2*R*_0_ + *z*_0_]*/c* is produced by combining the area *As* with the attenuation 1*/*[*4πct*] and the pulse height *α*:

26 $$\left| \varphi \right| = \frac{\alpha As}{{4\pi ct}} = \frac{{\alpha 2\pi R_{0} t\varepsilon c^{2} }}{{4\pi ct\left[ {R_{0} + z_{0} } \right]}} = \frac{{R_{0} c\varepsilon \alpha }}{{2\left[ {R_{0} + z_{0} } \right]}}$$

Because *cεα* = 1

27 $$\left| \varphi \right| = \frac{{R_{0} }}{{2\left[ {R_{0} + z_{0} } \right]}}$$

which holds for *z*_0_ ≤ *ct* ≤ [2*R*_0_ + *z*_0_]*.* (See Supplementary Note A for a different derivation) For fixed *z*_0_, |*φ*| observed at *P* is a rectangular pulse in time having temporal width 2*R*_0_*/c*. Note that as *z*_0_* → *0 or *R*_0_* → ∞*, |*φ*|→ 1*/*2, which is the same as for the planar source.

The wave field *φ* observed at *P* as a function of time is produced by using Heaviside step functions to implement the time dependence, *z*_0_ ≤ *ct* ≤ [2*R*_0_ + *z*_0_]:

28 $$\varphi \left( {z_{0} ,t} \right) = \left| \varphi \right|\left[ {\theta \left( {ct - z_{0} } \right) - \theta \left( {ct - \left[ {z_{0} + 2R_{0} } \right]} \right)} \right] = \frac{{R_{0} }}{{2\left[ {R_{0} + z_{0} } \right]}}\left[ {\theta \left( {ct - z_{0} } \right) - \theta \left( {ct - \left[ {z_{0} + 2R_{0} } \right]} \right)} \right]$$

**Figure 6:**
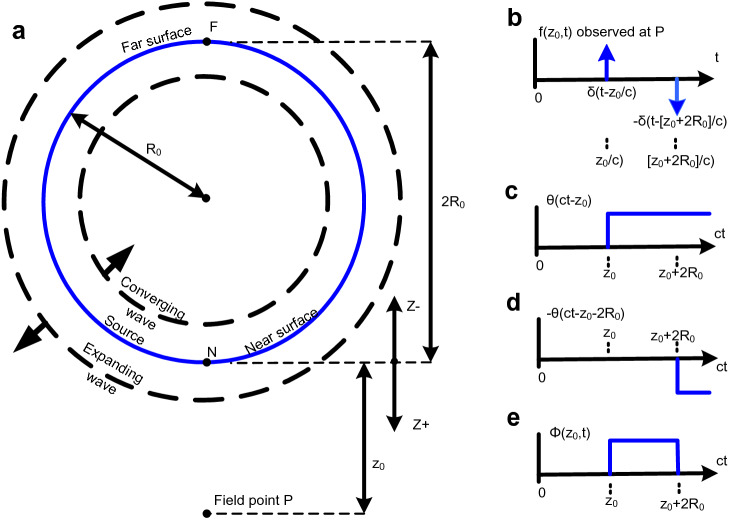
Radiated wave fronts and individual terms in the equations. The factor *R*_0_*/*[2[*R*_0_ + *z*_0_]] is not included in the figures' annotations. (**a**) A spherical source of radius *R*_0_ with expanding and converging wave fronts. The distance *z*_0_ is from the surface of the spherical source to the field point *P* at which the wave fronts will be observed. The two points N and F are the nearest and furthest points from *P* on the near and far surfaces of the spherical source. (**b**) Shows the impulses from the equation for the wave front *f*(*z*_0_,*t*): the impulse *δ*(*t* *−* *z*_0_*/c*) observed at *P*, at distance z_0_, is caused by an expanding impulsive spherical wave. This is the forward wave front for the spherical source from its near surface. The impulse *−* *δ*(*t* *−* [*z*_0_ + 2*R*_0_]*/c*) is caused by an impulsive spherical wave front, observed at *P*, that had initially converged with decreasing radius inward toward the spherical source's center, passed through the center while changing sign, and then expanded with increasing radius toward *P*. This is the backward wave front for the spherical source from the far surface. (**c**) The wave field corresponding to the forward wave front. (**d**) The wave field corresponding to the backward wave front. (**e**) The combined wave field resulting from the summation of the forward and backward wave fields.

The derivative of *φ* yields the wave front observed at *P* as a function of time (Fig. 6b):

29 $$\begin{gathered} f\left( {z_{0} ,t} \right) = \frac{{\partial \varphi (z_{0} ,t)}}{\partial t} = \frac{{cR_{0} }}{{2\left[ {R_{0} + z_{0} } \right]}}\left[ {\delta \left( {ct - z_{0} } \right) - \delta \left( {ct - z_{0} - 2R_{0} } \right)} \right] \\ = \frac{{cR_{0} }}{{2\left[ {R_{0} + z_{0} } \right]}}\left[ {\delta \left( {c(t - \frac{{z_{0} }}{c})} \right) - \delta \left( {c(t - \frac{{z_{0} + 2R_{0} }}{c})} \right)} \right] \, \\ = \frac{{cR_{0} }}{{2\left[ {R_{0} + z_{0} } \right]}}\left[ {\frac{1}{c}\delta \left( {t - \frac{{z_{0} }}{c}} \right) - \frac{1}{c}\delta \left( {t - \frac{{z_{0} + 2R_{0} }}{c}} \right)} \right] \\ = \frac{{R_{0} }}{{2\left[ {R_{0} + z_{0} } \right]}}\left[ {\delta \left( {t - \frac{{z_{0} }}{c}} \right) - \delta \left( {t - \frac{{z_{0} + 2R_{0} }}{c}} \right)} \right] \, \\ \end{gathered}$$

For finite *z*_0_ and *t* in the equation for *f*(*z*_0_,*t*), as *R*_0_* → ∞* the argument of *δ*(*ct* *−* *z*_0_ *−* 2*R*_0_) never equals zero, so never ‘activates’ and *δ*(*ct* *−* *z*_0_ *−* 2*R*_0_) may be ignored. Then

30 $$f\left( {z_{0} ,t} \right) = \frac{{R_{0} }}{{2\left[ {R_{0} + z_{0} } \right]}}\delta \left( {t - \frac{{z_{0} }}{c}} \right) = \frac{1}{2}\delta \left( {t - \frac{{z_{0} }}{c}} \right) \, as \, R_{0} \to \infty$$

which is the same as the equation for the planar source advancing wave front.

Both propagating spherical wave fronts (Fig. 6a) are affected by the spherical spreading term *R*_0_*/*[*R*_0_ + *z*_0_] which supplies the additional attenuation due to the radius increasing from the initial *R*_0_ to *R*_0_ + *z*_0_ (it is implied that spherical spreading attenuation is included in the initial strength of the wavelets originating on the sphere's surface). For example, [1*/R*_0_] [*R*_0_*/*[*R*_0_ + *z*_0_]] = 1[(*R*_0_ + *z*_0_]. In *−* *δ*(*t* *−* [*z*_0_ + 2*R*_0_]*/c*) the inverse spreading due to going from the far surface to the center is exactly cancelled by the spreading resulting from going from the center to the near surface. So the same *R*_0_*/*[*R*_0_ + *z*_0_] spherical spreading term can ultimately be applied to both the near and far surface waves that are to be observed at *P*.

The wave field *θ*(*ct* *−* *z*_0_ *−* 2*R*_0_) originated as a positive amplitude propagating wave field on the far surface (in the limit as R_0_ → ∞ the wave fields close to the surface of the sphere must be the same as the wave fields from the plane, and the wave fields on both sides of the plane are positive). As the initially converging spherical wave field passes through the center its amplitude changes sign (Gouy phase shift^39–41^: "It is well known that a spherical converging light wave undergoes a phase change of 180 degrees in passing through its focus…and is, in fact. a general property of any focused wave.", Boyd p. 877)^40^, and see Fig. 6d.

The wave field then begins expanding. When it expands through the surface of the spherical source the now negative wave field cancels the remainder of the wave field that originated on the near surface, *θ*(*ct* *−* *z*_0_), (Fig. 6c). The combined wave field (Fig. 6e) when observed in time at *P* is a rectangular pulse of time duration 2*R*_0_*/c* .

Notice that only the two points *N* and *F* (Fig. 6a), contribute as sources to the wave front *f*(*z*_0_,*t*). This is because *φ*(*z*_0_, *t*) is a rectangular pulse of temporal width 2*R*_0_*/c* for fixed *z*_0_ and its derivative is zero except on its leading and trailing edges (which originated at the two points *N* and *F*). Because the derivative is zero there is no wake (except for an impulse corresponding to the trailing edge). Also there is a one to one correspondence between the points on the source and the points on the wave front.

Therefore a spherical distribution of point sources radiating impulsive spherical wavelets creates two impulsive spherical wave fronts (Fig. 6b). Both propagate cleanly without a wake. Again this shows that the Huygens' wavelets cancel each other when summed together and differentiated, except where they make contact with their tangent surfaces.

The same 1/2 factor occurs in *f* as occurred in the case of the stationary infinite planar source. Note also there are forward (expanding) and backward (converging) wave fronts. When the sphere's radius expands with a speed equal to the propagation speed, the 1/2 factor and the backward wave both disappear, as will be shown.

### Expanding spherical source and elimination of the backward wave

This section analyzes the changes that occur in the results of section “Non expanding spherical source and elimination of the wake” when the spherical source is expanding with radial speed v. It is shown that the results for the non expanding sphere in section “Non expanding spherical source and elimination of the wake” can be used if the effects of motion on the Huygens' wavelets are included.

**Figure 7:**
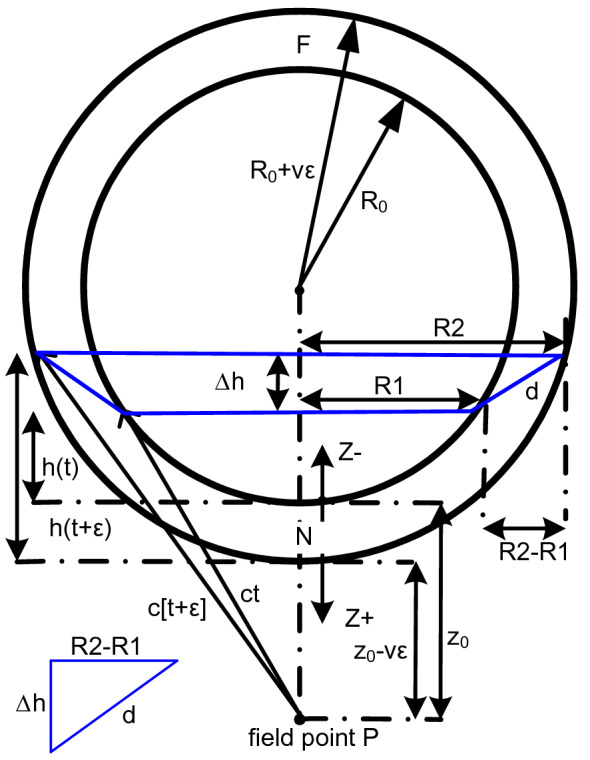
The impulsive spherical shell source (the 'sphere') with initial radius *R*_0_ expanding with a positive relative radial speed *v* where as with the planar source, *v* will be positive for forward wave fronts (in the direction of the expansion) and will be proceeded by a negative sign for backward wave fronts. *R*1 is the radius of the circle created by the intersection at time *t* of a sphere centered at *P* of radius *ct* with the spherical source. *R*2 is the radius of the circle created by the intersection at time *t* + *ε* of a sphere centered at *P* of radius *c*[*t* + *ε*] with the spherical source.

When this is done it is seen that a new additional wave is created. (A similar additional wave is discussed in section “Moving planar source and elimination of the backward wave”.) The amplitude of this additional wave is a function of the radial expansion speed, v. This additional wave reduces the amplitude of the backward wave and increases the amplitude of the forward wave. When v = c the backward wave is eliminated and the forward wave amplitude is doubled. In any case the original wave is replicated and there is no wake. See Fig. 7.

See Fig. 7. During the time interval *ε* the spherical source radius expands a distance *vε*. Similar to the case of the moving planar source, *R*2 is the radius of the base of a right circular cone of slant height *c*[*t* + *ε*] and *R*1 is the radius of the base of a right circular cone of slant height *ct*. These two bases, *R*1, *R*2, define a conical frustum of height *Δh* and slant height *d*. The lateral area of this frustum, *π*[*R*2 + *R*1]*d*, is the locus of all radiating points which can contribute to the field at the point *P* during the time interval [*t*, *t* + *ε*].

The expanding equivalent, *As*_*v*_, of the nonexpanding spherical zone area, *As*, must be derived. This is the lateral area of the frustum.

The frustum area is

31 $$As_{v} = \pi \left[ {R1 + R2} \right]d$$

where the slant height is

32 $$d^{2} = \Delta h^{2} + \left[ {R2 - R1} \right]^{2}$$

and the height is

33 $$\Delta h = h(t + \varepsilon ) - h(t) - v\varepsilon$$

The height h(t) was found previously as

$$h(t) = \frac{{c^{2} t^{2} - z_{0}^{2} }}{{2\left[ {R_{0} + z_{0} } \right]}}$$

that plus the two right triangles T1 and T2 can be solved for h(t + ε):

34 $${\text{T1: }}c^{2} \left[ {t + \varepsilon } \right]^{2} = R2^{2} + \left[ {z_{0} - v\varepsilon + h(t + \varepsilon )} \right]^{2}$$

35 $${\text{T2: }}\left[ {R_{0} + v\varepsilon } \right]^{2} = R2^{2} + \left[ {R_{0} + v\varepsilon - h(t + \varepsilon )} \right]^{2}$$

36 $${\text{T1 - T2: }}c^{2} \left[ {t + \varepsilon } \right]^{2} - \left[ {R_{0} + v\varepsilon } \right]^{2} = \left[ {z_{0} - v\varepsilon + h(t + \varepsilon )} \right]^{2} - \left[ {R_{0} + v\varepsilon - h(t + \varepsilon )} \right]^{2}$$

Expanding the terms on the left side of T1–T2 gets:

37 $$\begin{gathered} c^{2} \left[ {t + \varepsilon } \right]^{2} - \left[ {R_{0} + v\varepsilon } \right]^{2} = c^{2} t^{2} + 2c^{2} t\varepsilon + c^{2} \varepsilon^{2} - R_{0}^{2} - 2R_{0} v\varepsilon - v^{2} \varepsilon^{2} \\ = c^{2} t^{2} + 2c^{2} t\varepsilon - R_{0}^{2} - 2R_{0} v\varepsilon {\text{ as }}\varepsilon \to {0} \\ \end{gathered}$$

Expanding the two terms on the right side of T1–T2 yields for the first term:

38 $$\left[ {z_{0} - v\varepsilon + h(t + \varepsilon )} \right]^{2} = h^{2} (t + \varepsilon ) + z_{o}^{2} + v^{2} \varepsilon^{2} + 2h(t + \varepsilon )z_{0} - 2z_{0} v\varepsilon - 2h(t + \varepsilon )v\varepsilon$$

and yields for the second term:

39 $$\left[ {R_{0} + v\varepsilon - h(t + \varepsilon )} \right]^{2} = R_{0}^{2} + v^{2} \varepsilon^{2} + h^{2} (t + \varepsilon ) + 2R_{0} v\varepsilon - 2v\varepsilon h(t + \varepsilon ) - 2R_{0} h(t + \varepsilon )$$

Simplifying and subtracting the two terms, yields for the right side of T1–T2:

40 $$z_{0}^{2} - R_{0}^{2} + 2h(t + \varepsilon )z_{0} - 2R_{0} v\varepsilon - 2z_{0} v\varepsilon + 2R_{0} h(t + \varepsilon )$$

Equating the left side to the right side of T1–T2 produces

41 $$c^{2} t^{2} + 2c^{2} t\varepsilon - R_{0}^{2} - 2R_{0} v\varepsilon = z_{0}^{2} - R_{0}^{2} + 2h(t + \varepsilon )z_{0} - 2R_{0} v\varepsilon - 2z_{0} v\varepsilon + 2R_{0} h(t + \varepsilon )$$

42 $$\begin{gathered} c^{2} t^{2} + 2c^{2} t\varepsilon = z_{0}^{2} + 2h(t + \varepsilon )z_{0} - 2z_{0} v\varepsilon + 2R_{0} h(t + \varepsilon ) \\ = z_{0}^{2} - 2z_{0} v\varepsilon + 2h(t + \varepsilon )\left[ {R_{0} + z_{0} } \right] \\ \end{gathered}$$

Solving for *h*(*t* + *ε*) results in:

43 $$h(t + \varepsilon ) = \frac{{c^{2} t^{2} - z_{0}^{2} + 2c^{2} t\varepsilon - 2z_{0} v\varepsilon }}{{2\left[ {R_{0} + z_{0} } \right]}}$$

Then

44 $$\Delta h = h(t + \varepsilon ) - h(t) - v\varepsilon = \frac{{2c^{2} t\varepsilon - 2z_{0} v\varepsilon }}{{2\left[ {R_{0} + z_{0} } \right]}} - v\varepsilon = \varepsilon \left[ {\frac{{c^{2} t - z_{0} v}}{{\left[ {R_{0} + z_{0} } \right]}} - v} \right]$$

and squaring both sides and taking a limit gets

45 $$\Delta h^{2} = \varepsilon^{2} \left[ {\frac{{c^{2} t - z_{0} v}}{{\left[ {R_{0} + z_{0} } \right]}} - v} \right]^{2} = 0{\text{ , as }}\varepsilon \to {0}$$

So the frustum area as ε → 0 is

46 $$As_{v} = \pi \left[ {R1 + R2} \right]d = \pi \left[ {R1 + R2} \right]\sqrt {\Delta h^{2} + (R2 - R1)^{2} } = \pi \left[ {R1 + R2} \right]\left[ {R2 - R1} \right] = \pi \left[ {R2^{2} - R1^{2} } \right]$$

which is also the area, *Ap*, of an annulus with radii *R*1 and *R*2.

This annulus area *Ap* = 2*πc*^2^*tε* was previously computed for the stationary planar source and was also used in determining the area of the nonexpanding spherical source, *As* = [*R*_0_*/*[*R*_0_ + *z*_0_]]2*πc*^2^*tε*. Consequently the previously developed results for the non expanding sphere can be used if the effects of the motion of the expanding spherical source on its wavelet point sources' initial impulses are included:

At the wavelet point source location as *z*_0_* → *0 the motion due to the sphere's expanding radius is the same as the motion of the moving planar source if spherical spreading is disregarded. Then the method used for the moving planar source will be used here to implement the effects of motion on the wavelet point sources initial impulses due to the sphere's radius expanding with a speed *v*:

As with the non expanding sphere, the wavelet point sources at points N and F of the near and far surfaces of the expanding sphere are the sole contributors to the wave front along the radial line. The wavelet point sources at points N and F on the near and far surfaces have relative speeds + *v* and *−* *v* with respect to their originating surfaces. (The far surface is the source for a backward propagating wave front and so has relative speed *−* *v*.)

Including the moving planar source's source speed effect in the equation for the wave front from a nonexpanding spherical source observed at *P* as a function of time yields:

47 $$f\left( {z_{0} ,t} \right) = \frac{{R_{0} }}{{2\left[ {R_{0} + z_{0} } \right]}}\left[ {\frac{v + c}{c}\delta \left( {t - \frac{{z_{0} }}{c}} \right) - \frac{ - v + c}{c}\delta \left( {t - \frac{{z_{0} + 2R_{0} }}{c}} \right)} \right]$$

for the forward propagating spherical wave front from the near surface combined with the backward propagating spherical wave front from the far surface of the expanding spherical source.

When source speed equals zero, *v* = 0, the result is the same as for the non expanding spherical source.

When source speed equals the propagation speed, *v* = *c*, the equation becomes

48 $$f\left( {z_{0} ,t} \right) = \frac{{R_{0} }}{{2\left[ {R_{0} + z_{0} } \right]}}\left[ {\frac{c + c}{c}\delta \left( {t - \frac{{z_{0} }}{c}} \right) - \frac{ - c + c}{c}\delta \left( {t - \frac{{z_{0} + 2R_{0} }}{c}} \right)} \right] = \frac{{R_{0} }}{{R_{0} + z_{0} }}\delta \left( {t - \frac{{z_{0} }}{c}} \right)$$

So when *v* = *c* the backward wave front is eliminated and *f*(*z*_0_,*t*) yields an expanding forward propagating impulsive spherical wave front, which is an impulse when seen at *P* as a function of time. (Note the absence of the ½ factor). Since this holds for every radial line, this is the new impulsive spherical progressing Huygens' wave front which has neither a wake nor a backward wave.

The spherical spreading factor $\frac{{R_{0} }}{{R_{0} + z_{0} }}$ differentiates this result from the result from the moving planar source and accounts for the radial lines representing infinitesimal solid angles. As *R*_0_* → ∞* this equation becomes the same as for the planar source moving with speed equal to *c*.

### Huygens' Principle as depicted in his figures

It was shown that for the spherical and planar source, when the excitation is *δ*(*t*), the resulting wave field *ϕ* when differentiated disappears except at the locations of the propagating wave fronts *f*. The following shows how the intermediate step of differentiation can be eliminated.

Let *L* represent the linear wave propagation operator which transforms the excitation on either the plane or sphere into the wave field *ϕ*, If *δ*(*t*) is the excitation, the resulting wave field due to the wave propagation process is

49 $$\phi \left( {z,t} \right) = L\left( {\delta \left( t \right)} \right)$$

which is the response of *L* to an impulse. As a function of time in the case of the plane this wave field is a step function and in the case of the sphere it is a rectangular pulse.

Because *L* is linear (LTI) the derivative can be taken of both *ϕ* and of *δ*(*t*)^42^ yielding

50 $$\frac{\partial }{\partial t}\phi \left( {z,t} \right) = L\left( {\frac{d}{dt}\delta \left( t \right)} \right) = L\left( {\delta^{\prime}\left( t \right)} \right)$$

But the derivative of the wave field *ϕ* is the wave front *f*, so

51 $$f(z,t) = \frac{\partial }{\partial t}\phi \left( {z,t} \right) = L\left( {\delta^{\prime}\left( t \right)} \right)$$

As a result, if the doublet *δ′*(*t*) is used as the initial excitation instead of the singlet *δ*(*t*) for the wavelets originating from the source, the Huygens' wavelets when combined disappear except where they contact their common tangent surfaces. Also when the sources' motion equals the wave propagation speed the backward wave corresponding to the backward tangent surface disappears. The remaining forward tangent surface is the new propagating wave front, obtained without the intermediate step of differentiation.

In Huygens' figures the wavelets then would represent doublets [*4πr*]^*−*1^*δ′*(*t* *−* *r/c*). However, notice that the resulting new wave fronts are of the form *δ* not *δ′* so they must be differentiated before being used as the initial excitation in the next iteration of wave propagation.

Because *ϕ* is the response to an impulse, the analysis can be applied to other forms of excitation, say *g*(*t*), by using convolution: the convolution

52 $$L(g) = g * L(\delta ) = g * \phi$$

gives the response of *L* to the excitation *g*.

## Summary

Huygens' geometrical construction has been shown to be literally correct as depicted in his figures (see Fig. 1a) when his wavelets represent the first derivative of Dirac delta distributions and the source motion is equal to the propagation speed. For the sphere and the infinite plane there is no wake and there is no backward wave, thus solving the two most long standing problems (1678).

An intuitive, relatively simple method to analyze wave propagation has been provided which provides a clear view of the propagation process starting with the very first wave propagation analysis, Huygens' Principle. This will facilitate the teaching of wave physics and provide a new model for wave propagation.

## Supplementary Information

Supplementary Information.
