# Supplementary material for: Huygens' Principle geometric derivation and elimination of the wake and backward wave
Source: Sci Rep. 2021 Oct 12;11:20257. doi: 10.1038/s41598-021-99049-7 (PMC8511121; doi:10.1038/s41598-021-99049-7)
Supplement: Supplementary file 1 — Supplementary Information. [file 41598_2021_99049_MOESM1_ESM.doc]

**Supplementary Information**

# Huygens' Principle geometric derivation and elimination of the wake and backward wave

Forrest Anderson

**Supplementary Notes**

# **Supplementary Notes**

***Note A ) Derivation of the spherical source's wave field due to an impulsive excitation using integration***

The following uses these references.

For the response to an impulse see:

Brian Moths (https://physics.stackexchange.com/users/23785/brian-moths), What is the wave propagated away from an impulsively excited spherical shell?, URL (version: 2015-01-21): https://physics.stackexchange.com/q/160573

For the surface element see:

Andrei (https://math.stackexchange.com/users/331661/andrei), Confused with a spherical coordinate system surface element, URL (version: 2019-04-24): https://math.stackexchange.com/q/3201021

**Figure A1** shows the spherical source's geometry and nomenclature (different from the main body text) for the case of **r_S_** lying in the XY plane which is perpendicular to *Z*. R is the sphere's radius, S is the spherical source, and the wave field observation point is on the z axis at z_0_. **r_0_** is a vector from the origin to that point. **r_S_** is a vector from the origin to the impulsive wavelet source point under consideration on the spherical source. *D* is the distance between these two vectors. For convenience define $\alpha\equiv\sqrt{R^{2}-z_{S}^{2}}=\sqrt{D^{2}-\left(z_{0}-z_{S}\right)^{2}}$ . **(a)** View down the Y axis. **(b)** View down the Z axis.

The field at **r_0_** is found by summing contributions from the impulsive wavelets from every point on the spherical source:

(A1)

The delta distribution's argument, *D-ct*, activates the impulse at time *t = D/c* at the observation point *z_0_*.

Choosing coordinates: Because of symmetry around the z axis **r_0_** = (0, 0, z_0_). Also . where *ϕ* is the angle of **r**_s_ in the XY plane and α is the length of **r**_s_ in the XY plane. 1/(4πD) implements the spherical spreading for the wavelets.

Then

(A2)

So *D* is a function of *z_s_* with parameters *R* and *z_0_* :

$D\equiv D(z_{S})=\sqrt{R^{2}-z_{S}^{2}+\left(z_{0}-z_{S}\right)^{2}}$ (A3)

Then we must find the equation for the surface element corresponding to d**r_S_** : The surface element can be derived from the volume element where *J* is the Jacobian of the transformation from (x.y,z) to (r,z,ϕ). The surface element is perpendicular to the sphere's radius vector **r** so .

The Jacobian is

(A4)

$$=aei+bfg+cdh+-ceg-bdi-afh$$

Because *c = i = 0* and also because *f = 1,* the Jacobian is *J = bg - ah* which yields

(A5)

Setting |J| = |-*r*| *= R* yields *ds = Rdzdϕ* .

Noting that *dz* is *dz_S_*, and recognizing that integration over *S* can done by letting *z_S_* range from $-R\text{ to}+R$, the integral becomes

(A6)

Integrating over *ϕ* yields

$\psi\left(z_{0},t\right)=2\pi R\int_{-R}^{+R} \frac{1}{4\pi D}\delta\left(D-ct\right)dz_{S}=\frac{R}{2}\int_{-R}^{+R} \frac{1}{D}\delta\left(D-ct\right)dz_{S}$ (A7)

Note that *2πRdz_S_* is the area of a spherical zone of height *dz_S_* , so the integral is summing over spherical zones.

In preparation for using a change of variable $u=D-ct$ compute the derivative with respect to *z_S_* of *u,* the argument of the delta function:

$\begin{matrix} \frac{du}{dz_{S}}=\frac{\partial}{\partial z_{S}}\left(D-ct\right)=\frac{\partial}{\partial z_{S}}D=\frac{\partial}{\partial z_{S}}\sqrt{R^{2}-z_{S}^{2}+(z_{0}-z_{S})^{2}} \\ =\frac{1}{2\sqrt{R^{2}-z_{S}^{2}+(z_{0}-z_{S})^{2}}}\frac{\partial}{\partial z_{S}}\left(R^{2}-z_{S}^{2}+(z_{0}-z_{S})^{2}\right) \\ =\frac{1}{2D}\frac{\partial}{\partial z_{S}}\left(R^{2}-z_{S}^{2}+z_{0}^{2}-2z_{0}z_{S}+z_{S}^{2}\right) \\ =\frac{1}{2D}\frac{\partial}{\partial z_{S}}\left(-2z_{0}z_{S}\right)=\frac{-2z_{0}}{2D}=\frac{-z_{0}}{D} \end{matrix}$ (A8)

So $du=\frac{-z_{0}}{D}dz_{S}\text{ ; and }dz_{S}=\frac{-D}{z_{0}}du$

Then change the integral limits from being in terms of *z_S_* to being in terms of *u* so that the integral runs from the nearest to the furthest points on *S*. From the geometry the upper limit $z_{S}=+R\text{ becomes }u=ct-[z_{0}+R]\text{ }$and the lower limit.

With this change of variables the integral becomes

$\begin{matrix} \psi(z_{0},t)=\frac{R}{2}\int_{-R}^{+R} \frac{1}{D}\delta\left(D-ct\right)dz_{S}=\frac{R}{2}\int_{ct-(z_{0}-R)}^{ct-(z_{0}+R)} \frac{1}{D}\delta(u)\frac{-D}{z_{0}}du \\ =\frac{-R}{2z_{0}}\int_{ct-(z_{0}-R)}^{ct-(z_{0}+R)} \delta(u)du=\frac{R}{2z_{0}}\int_{ct-(z_{0}+R)}^{ct-(z_{0}-R)} \delta(u)du \\ =\frac{R}{2z_{0}}\theta(u)|_{ct-(z_{0}+R)}^{ct-(z_{0}-R)} =\frac{R}{2z_{0}}[\theta(ct-(z_{0}-R))-\theta(ct-(z_{0}+R))] \end{matrix}$ (A9)

Therefore

$\psi(z_{0},t)=\frac{R}{2z_{0}}\text{ when }\left|z_{0}-R|<ct<\right|z_{0}+R|\text{, else it is zero}$ (A10)

Converting to the variables used in the main text where *R* here equals *R_0_* there and ***z_0_*** here equals *R_0_* ***+ z_0_*** there, we get for the field :

$\psi(z_{0},t)=\frac{R_{0}}{2(R_{0}+z_{0})}\text{ when z}_{0}\leq ct\leq z_{0}+2R_{0}\text{ , else it is zero}$ (A11)

This is the same as in the main body text. ( Note that in the main body text the symbol ϕ is used for the field )

***Note B ) Derivation of the additional pulses due to the impulsive planar source motion using D'Alembert's formula, and the correspondence with the geometrical derivation***

D'Alembert's formula [2, 3] for the solution to the one dimensional wave equation is

$u(x,t)=\frac{f(x+ct)+f(x-ct)}{2}+\frac{1}{2c}\int_{x-ct}^{x+ct} g(s)ds$ (B1)

where *f(x)* and *g(x)* are the initial conditions for displacement and speed of displacement at *t = 0*. The integral term which gives the displacement resulting from an initial speed of displacement is

$u(x,t)=\frac{1}{2c}\int_{x-ct}^{x+ct} g(s)ds\text{ , where }g(x)=u_{t}(x,0)$ (B2)

(See Supplementary Note E) In addition to its normal use, the integral term will be used in the following to get the additional displacement pulses due to an initial speed of the original source.

For a very small integration interval around *x* the integral term can be simplified: Let the spatial interval width be $(x+c\Delta t)-(x-c\Delta t)=2c\Delta t$. Also *g(s)* is approximately constant in the interval so D'Alembert's formula's integral term reduces to

(B3)

which gives the displacement resulting from the displacement speed pulse of temporal width *2Δt* passing across a point, *x*. ( In the factor preceding the integral the *1/c* factor converts the integral results, *g(x)2cΔt*, into displacement units. The *1/2* factor accounts for the splitting into the forward and backward waves. )

So for a very small integration interval centered at *x* the integral term giving displacement at *x* is approximately the product of the temporal integration interval and the displacement speed evaluated at *x*. This is used in the following to derive the additional pulses due the impulsive planar source constant motion, *v*:

The temporal width of the pulse arriving from the moving impulsive planar source is *ε* which determines the temporal width of the additional pulse and so determines the effective temporal integration interval. Also the displacement speed *g(x)* is equal to the value of *v*, a constant, which is the speed of the planar source, so the spatial integration interval is *vε* . (The direction of *v* can be parallel (longitudinal) or orthogonal (transverse) to the direction of propagation) Then the spatial extent of the additional pulse is *vε* in either the transverse or longitudinal direction.

The additional pulse's displacement height is *α/2*, which is determined by the height of the original pulse arriving from the moving impulsive planar source. Note that *α* is *1/cε*. Then the strength of the additional pulse in the forward direction is the product of the spatial extent and the height:

$v\varepsilon\frac{\alpha}{2}=\frac{v\varepsilon}{2c\varepsilon}=\frac{v}{2c}$ (B4)

As *ε → 0* a pulse of width *ε* and height *1/ε* moving at speed *c* tends to *δ(x-ct)*. So as *ε → 0* the additional pulse becomes an impulse with strength *v/[2c].* Then the additional pulse in the forward direction as a function of time is

$\frac{v}{2c}\delta(t-x/c)$ (B5)

In the backward direction the sign in front of *v* is negative because the displacement speed pulse propagates in the direction opposite to the wavefront. Then the strength of the additional pulse in the backward direction is

$-v\varepsilon\frac{\alpha}{2}=\frac{-v\varepsilon}{2c\varepsilon}=\frac{-v}{2c}$ (B6)

This gives the additional pulse in the backward direction as *ε → 0* :

$\frac{-v}{2c}\delta(t+x/c)$ (B7)

**Figure B1** The additional pulses. **(a)** The forward additional pulse. **(b)** The backward additional pulse.

So there is an exact correspondence between the geometrical and D'Alembert derivations of the additional pulses caused by an impulsive planar source speed equal to *v*.

***Note C ) Cancellation of backward wave using D'Alembert's formula, and correspondence with the geometrical derivation when v = c***

Given the one dimensional wave equation

$\frac{d^{2}u(x,t)}{dx^{2}}-\frac{1}{c^{2}}\frac{d^{2}u(x,t)}{dt^{2}}=0$ (C1)

with initial conditions:

$\begin{matrix} u(x,0)\equiv f(x) \\ \frac{\partial u(x,t)}{\partial t}|_{t=0} \equiv g(x) \end{matrix}$ (C2)

The solution is given by D'Alembert's formula [2, 3] (See Supplementary Note E):

$u(x,t)=\frac{f(x+ct)+f(x-ct)}{2}+\frac{1}{2c}\int_{x-ct}^{x+ct} g(y)dy$ (C3)

If *G(y)* is defined as the antiderivative:

$G(y)\equiv\int g(y)dy$ (C4)

then

$u(x,t)=\frac{f(x+ct)+f(x-ct)}{2}+\frac{1}{2c}\left[G(x+ct)-G(x-ct)\right]$ (C5)

In view of this equation, for no backward wave it is required that:

$\begin{matrix} G(x-ct)=-cf(x-ct) \\ G(x+ct)=-cf(x+ct) \end{matrix}$ (C6)

Using these values

$u(x,t)=\frac{f(x+ct)+f(x-ct)}{2}+\frac{1}{2c}\left[-cf(x+ct)+cf(x-ct)\right]=f(x-ct)$ (C7)

The backward wave is cancelled and the ½ factor in the forward wave disappears.

Note that the last two terms, *-½f(x+ct)* and *+½f(x-ct),* are the additional pulses created by the integral term in D'Alembert's formula when v is equal to the propagation velocity ( see equations B5 and B7 with *v = c* ). This is the same as the geometrical derivation.

To achieve this it is needed that *G(x) = -cf(x)* at *t = 0* so that the initial conditions satisfy

$g(x)=\frac{\partial G(x,t)}{\partial x}|_{t=0} =-c\frac{df(x)}{dx}$ (C8)

See Bland [1] (p. 63, 64) for a different derivation.

Then we get

$\int_{x-ct}^{x+ct} g(y)dy=-c[f(x+ct)-f(x-ct)]=-cf(x+ct)+cf(x-ct)$ (C9)

as required.

For an example, let the initial displacement be

(C10)

then

(C11)

and

(C12)

so

$G(x)=-c\delta(x)$ (C13)

Then

$u(x,t)=\frac{\delta(x+ct)+\delta(x-ct)}{2}+\frac{1}{2c}\left[-c\delta(x+ct)+c\delta(x-ct)\right]=\delta(x-ct)$ (C14)

Again, the backward wave is cancelled and the ½ factor in the forward wave disappears.

***Note D ) Cancellation of the backward wave in an ongoing propagating one dimensional impulsive wave field by using the integral term in D'Alembert's formula***

Consider an ongoing wave analyzed by examining propagation of a right moving impulse *δ(x - ct)* over a very small interval enclosing a point on the *x* axis There will be an incoming wave and an outgoing wave. The incoming wave will supply the initial conditions for the outgoing wave.

D'Alembert's formula [2, 3] for the solution to the 1D wave equation is

$u(x,t)=\frac{f(x+ct)+f(x-ct)}{2}+\frac{1}{2c}\int_{x+ct}^{x+ct} g(s)ds$ (D1)

Where *f(x)* and *g(x)* are the initial conditions for displacement and speed of displacement at *t = 0* and are given by

$$\begin{matrix} u(x,0)\equiv f(x) \\ \frac{\partial u(x,t)}{\partial t}|_{t=0} \equiv g(x) \end{matrix}$$

(D2)

The initial displacement condition, *f(x)*, at *t =* 0 due to the incoming impulsive wave that is moving to the right is

$f(x)=u(x,0)=\delta(x-ct)|_{t=0}=\delta(x)$ (D3)

The displacement due to that initial displacement condition would be

$\frac{1}{2}[f(x-ct)+f(x+ct)]=\frac{1}{2}[\delta(x-ct)+\delta(x+ct)]$ (D4)

However the initial speed of displacement condition, *g(x)*, at *t =* 0 can be derived in this case from the right moving impulse in the initial displacement condition since the incoming pulse is only right moving. Then also the ½ factor which accounts for splitting of the pulse disappears, so:

$g(x)=u_{t}(x,0)=\delta_{t}(x-ct)|_{t=0}=-c\delta'(x-ct)|_{t=0}=-c\delta'(x)$ (D5)

See Bland [1] (p. 63, 64) for a different derivation.

Then the displacement due to the initial speed of displacement of the outgoing wave can be derived by using these initial conditions in the integral term in D'Alembert's formula (see Supplementary Note E) which results in the additional pulses :

$\begin{matrix} \frac{1}{2c}\int_{x-ct}^{x+ct} g(y)dy=\frac{1}{2c}\int_{x-ct}^{x+ct} -c\delta'(y)dy \\ =-\frac{1}{2}[\delta(x+ct)-\delta(x-ct)]=\frac{1}{2}[\delta(x-ct)-\delta(x+ct)] \end{matrix}$ (D6)

Combining the displacements due to the initial displacement and due to the initial speed of displacement yields for the outgoing wave

$U(x,t)=\frac{\delta(x-ct)+\delta(x+ct)}{2}+\frac{\delta(x-ct)-\delta(x+ct)}{2}=\delta(x-ct)$ (D7)

The negative term cancels the left moving backward wave, and the remaining terms double the right moving forward wave eliminating the *1/2* factor. Note that if the incoming wave is left moving, the sign on *g(x)* changes and the right moving backward wave is canceled, and again the remaining terms double the left moving wave eliminating the *1/2* factor.

Then for an ongoing wave, at each point on the wave, the initial speed of displacement condition derived from the incoming wave creates the initial conditions for the outgoing wave needed to continue propagating the wave in the ongoing direction without continuously radiating backward waves and also without incurring attenuation from the *1/2* factor.

***Note E) D'Alembert formula integral term physical interpretation and geometric derivation***

***Figure E1*** shows a speed of displacement pulse, *g(x,t)* initially applied at *t = 0* while centered at *x = 0*, after it has propagated a short time. ( Note that the direction of wave propagation and direction of displacement caused by speed of displacement are not necessarily the same. ) Similarly to a displacement pulse, the speed of displacement pulse has split in two with pulses propagating in both the left and right directions:  *½g(x+ct)* and *½g(x-ct)* .

The resulting displacement at *x_0_* is a function of how long the propagating speed of displacement pulse is applied at *x_0_* as it passes. After the pulse has passed *x_0_* the displacement it caused at *x_0_* persists. This is the reason the displacement at *x_0_* due to an initial speed of displacement depends on the whole interval *x_0_ - ct* to *x_0_ + ct*. In contrast, the displacement resulting from an initial displacement at *t = 0* only exists at the ends of the interval, *x_0_ - ct* and *x_0_ + ct*. It does not persist at *x_0_* after the pulse passes.

If the spatial length of the pulse is a differential *dx* then the length of time that the pulse is applied at *x_0_*, or any other point, *x*, that it passes, is *dx/c.* Since the initial pulse at *t = 0* splits into left and right moving pulses the amplitude of the pulse is *g(x)/2*. The resulting differential displacement is

(E1)

If the pulse is replaced by an extended function, the resulting displacement would be given by the sum of differential displacements:

(E2)

where *ct* is the furthest distance any point on the initial speed of displacement function at *t = 0* can reside away from *x* in either the left or right direction for it to have effect at *x* at time *t*.

This is the integral term in D'Alembert's formula [2, 3].

**References**

1. Bland, D. R. *Vibrating Strings* 63 (Routledge & Kegan Paul Ltd, London 1960)
2. Borrelli, R. L., Courtney, S. C. *Differential Equations* (John Wiley & Sons, 1996)
3. Farlow, S. J., *Partial differential Equations For Scientists And Engineers* (Dover 1993)
